# Influence of Different Nanometals Implemented in PMMA Bone Cement on Biological and Mechanical Properties

**DOI:** 10.3390/nano12050732

**Published:** 2022-02-22

**Authors:** Beata Świeczko-Żurek, Andrzej Zieliński, Dorota Bociąga, Karolina Rosińska, Grzegorz Gajowiec

**Affiliations:** 1 Faculty of Mechanical Engineering and Ship Technology, Gdańsk University of Technology, 11/12 Narutowicza Str., 80-233 Gdańsk, Poland; andrzej.zielinski@pg.edu.pl (A.Z.); grzgajow@pg.edu.pl (G.G.); 2 Division of Biomedical Engineering and Functional Materials, Institute of Materials Science and Engineering, Lodz University of Technology, 1/15 Stefanowskiego Str., 90-537 Lodz, Poland; dorota.bociaga@p.lodz.pl (D.B.); karolina.rosinska@dokt.p.lodz.pl (K.R.)

**Keywords:** bone cement, PMMA, nanosilver, nanocopper, nanonickel, antibacterial resistance, cytotoxicity, compression strength, wettability

## Abstract

Cemented arthroplasty is a common process to fix prostheses when a patient becomes older and his/her bone quality deteriorates. The applied cements are biocompatible, can transfer loads, and dampen vibrations, but do not provide antibacterial protection. The present work is aimed at the development of cement with antibacterial effectivity achieved with the implementation of nanoparticles of different metals. The powders of Ag, Cu with particles size in a range of 10–30 nm (Cu10) and 70–100 nm (Cu70), AgCu, and Ni were added to PMMA cement. Their influence on compression strength, wettability, and antibacterial properties of cement was assessed. The surface topography of samples was examined with biological and scanning electron microscopy. The mechanical properties were determined by compression tests. A contact angle was observed with a goniometer. The biological tests included an assessment of cytotoxicity (XTT test on human cells Saos-2 line) and bacteria viability exposure (6 months). The cements with Ag and Cu nanopowders were free of bacteria. For AgCu and Ni nanoparticles, the bacterial solution became denser over time and, after 6 months, the bacteria clustered into conglomerates, creating a biofilm. All metal powders in their native form in direct contact reduce the number of eukaryotic cells. Cell viability is the least limited by Ag and Cu particles of smaller size. All samples demonstrated hydrophobic nature in the wettability test. The mechanical strength was not significantly affected by the additions of metal powders. The nanometal particles incorporated in PMMA-based bone cement can introduce long-term resistance against bacteria, not resulting in any serious deterioration of compression strength.

## 1. Introduction

### 1.1. Characteristics of Bone Cements

The bone cements are usually divided into acrylic and phosphate-based cements [1,2,3,4]. Polymethyl methacrylate (PMMA), commonly known as bone cement, is widely used for implant fixation in various orthopedic and trauma surgery. In reality, “cement” is a misnomer because the word “cement” is used to describe a substance, that bonds two things together. However, PMMA acts as a space-filler, that creates a tight space, which holds the implant in contact with the bone. Bone cements have no intrinsic adhesive properties, but they rely instead on a close mechanical interlock between the irregular bone surface and the prosthesis [5]. Adhesion improvement of 50% at the PMMA bone cement-titanium implant interface was obtained using atmospheric pressure plasma polymerization [6]. As concerns the phosphate-based cements, which usually are applied as bone-substitute materials, recently the brushite-based and monetite-based cements [1,7,8], those based on phosphate with bioactive ions, like Mg, Sr, Zn, Mn, Cu, Li, Co, Ag, [9], and silicate bone cements were proposed.

PMMA bone cements are two-component materials—powder (monomer) and liquid (hardener). After mixing these two components, they polymerize [10]. The polymerization process is accompanied by the release of heat, and as a result, the cement temperature can rise to even 96 °C after 6 min of mixing [11]. This results in tissue necrosis and, therefore, it is recommended to cool the prosthesis during implantation. The components of bone cement are sterilized with ethylene oxide or ionizing radiation (powder), as well as by infiltration (liquid). The addition of small amounts of the “radiosubstance” pacifying agent may lower the viscosity [12], and it is of great importance in the surgical process. Cement implanted under pressure is characterized by a very low viscosity, which is favorable for its fixation in the bone, but there is a risk of the monomer penetrating the bloodstream, and also of embolism [11]. Three types of cements are used: low, medium, and high viscosity. Low-viscosity cements have a long mixing phase and a short working phase, and high-viscosity cements—just opposite phase periods. In [12] one might find the opinion, that owing to the use of high-viscosity cement, a better fixation of the prosthesis can be obtained, than in the case of low-viscosity cement. With the increase in ambient temperature, the time of cement polymerization decreases, which means that the cement should be implanted in a shorter time.

The PMMA bone cements have some drawbacks as no bioactivity, no antibacterial protection, and excessive brittleness. Therefore, different additions to the base material were proposed as discussed below. Besides, to improve the effectiveness of the treatment for bone defects caused by metastatic bone tumors, the formulation of PMMA cement containing titania and magnetite that offers high bone affinity, making the cement suitable for use in magnetic hyperthermia, was developed [13].

### 1.2. Introducing the Bioactivity

To introduce the bioactivity, the increased cells’ proliferation was obtained for PMMA with carbon nanotubes (CNTs) and reduced graphene oxide (rGO) [14]. Other proposals of the same research team were PMMA, monticellite, and CNTs [15], and PMMA together with polycaprolactone (PCL), fluorapatite (FA), and graphene oxide (GO) [16]. The incorporation of multi-walled carbon nanotubes (MWCNTs) into PMMA bone cement improved cytocompatibility and osseointegration [17]. The addition of lactoferrin (LF) on the surface of solidified PMMA bone cement resulted in improving the adhesion, viability, proliferation, and differentiation of preosteoblasts [18]. The cell viability, growth, and cell adhesion increased for PMMA bone cement with 25% of chitosan/GO composite bone cement [19].

### 1.3. Introducing the Antibacterial Properties

To add an antibacterial barrier, usually, antibiotics were added to impregnate the cements. Such compositions are used routinely in endoprostheses revision. As the effectiveness of antibiotics on the bacterial flora cements produced in the factory is low, the surgeon often prepares cement-antibiotic mixtures during the surgery [12]. The antibiotic-impregnated cements remain a gold standard [20,21]. Among antibiotics usually applied for bone cements, are vancomycin [22], rifampicin [23], gentamycin [24,25], vancomycin, and gentamycin [26], cefazolin and gentamicin, and amphotericin against various bacteria and *Candida* biofilms [27], vancomycin, gentamycin and amphotericin [28], vancomycin, daptomycin, and tobramycin [29]. Antibiotic-loaded bone cements are effective by 20 to 84% to reduce the risk of prosthetic joint infection after implantation surgery [30]. In [31], the titanium dioxide (TiO_2_) nanotubes (TNTs) were formulated with antibiotic-loaded bone cement to enable an enhanced release of antibiotics, more than 50% of loaded antibiotics (such as gentamicin or vancomycin) were released in two months.

Another group of antibacterial substances is nanometals. Nanomaterials have unique physical-chemical properties, that differ significantly from those of the same materials on a micro and macro scale. These properties include a high surface area to mass ratio, high activity, and minimal diffusion restrictions. Nanoparticles significantly contributed to the development of pharmacotherapy, gene therapy, the development of modern drugs and methods of their delivery, and the improvement of imaging diagnostics techniques and other related fields [32]. The addition of nanomaterials can enhance the mechanical properties, fracture toughness if the application type, suitable process, loadings, size, type of nanomaterials are implemented appropriately, but if not, it may negatively influence the mechanical behavior [33]. The most applied is nanosilver [34]. In [35], the PMMA cement loaded with nanosilver at 1 wt.% fully opposed to different bacteria growth, more than gentamycin, at no cytotoxicity. Nanosilver was added as well as antibiotics [36] in bone cements. The positive influence of nanosilver and nanocopper on antibacterial efficiency was reported in [37,38], but also cytotoxicity caused by nanocopper. In [39], silver-containing bioactive glass powders were prepared by their introduction in the polymer matrix of cement with different viscosity. The silver nanoparticles capped with tiopronin conferred antimicrobial activity against methicillin-resistant *Staphylococcus aureus* (MRSA) at concentrations as low as 0.1 wt.% [40]. AgNPs at 0.25–1.0 wt.% introduced into the PMMA cement significantly reduced biofilm [35,41].

The inclusion of AuNPs at 1 wt.% into a PMMA-based bone cement was suggested to reduce the thickness of the biofilm and the ratio of live/dead bacterial cells [42]. Moreover, the carboxylic acid-functionalized polycarbonates were used to enhance the antibacterial performance of the bone cement without deterioration of mechanical strength and cellular biocompatibility [43]. Finally, the MgP nanosheets exhibited antibacterial properties against *Escherichia coli* [44].

### 1.4. Influence of Strengthening Additives on Mechanical Behavior

The mechanical properties may deteriorate following all admixtures added to bone cement. The reason for this negative impact can be either creating pores or inhibiting polymerization rate. Thorough mixing influences the even distribution of particles in the matrix, which affects the physical properties of the cement. During mixing, considerable amounts of air can be introduced, which results in porosity. These pores, as well as the pores formed by the evaporation of certain amounts of monomer inside the mass [12], may act as stress concentrators, reducing the cement resistance to cracking [10]. According to [12], maintaining the increased pressure during cement implantation ensured better bone penetration through the cement and thus improved the quality of the bone-cement interface, and also increased the fatigue strength of cement.

The compressive strength of bone cement, when it is used to fill the cavities should be at least 30 MPa, similar to that of naturally formed spongy bone, and at least 70 MPa, if used for fixing endoprostheses, [45]. The main mechanical tests of cement are compressive and bending strength tests—according to ISO and ASTM standards [46,47]. Conventional tests of the functional properties of bone cement mainly verify the polymerization process and basic mechanical properties, under the above standards. They include, apart from testing the cement polymerization process, determination of compressive strength, and bending strength, in a 3- or 4-point bending test, as well as the elastic modulus under bending conditions [48]. An impact test is also performed. Bone cement is characterized by about 2–3 times higher compressive strength, than tensile strength [49]. These properties are of particular importance for the durability of the bone-cement-denture connection in the conditions of the artificial joint operation. Cracks can develop more easily in areas of cement, that are subject to tensile stresses, than in areas of compressive stress. Bone cement is characterized by low impact strength [49,50]. Besides, various types of contaminants or additives as well as inadequate mixing and aging are factors affecting the mechanical properties of cement [51]. Different additives were added to cement to improve the mechanical properties. For example, strength values were observed to have improved by about 20% with 1.0 g of Pluronic^®^F127 (Poloxamer 407; polyol) [52].

### 1.5. Influence of Antibiotics on Mechanical Behavior

Concerning the antibiotics, their additions negatively affect the compressive and bending strengths of the cements [24]. For vancomycin-loaded cement [26,53], the strength value was decreased even below the ASTM F451 standard. The addition of gentamicin and amphotericin slightly decreased the compressive strength [27]. The mechanical properties were slightly decreased but if adding antibiotics at high contents [54]. Rifampicin deteriorated the mechanical properties of PMMA causing a delay in the PMMA polymerization [23]. The type of antibiotic was important for mechanical behavior: cefazolin-impregnated cements showed a lower strength than vancomycin-loaded ones [22].

### 1.6. Influence of Nanomaterials on Mechanical Behavior

Different factors are important for the mechanical properties of nanomaterials, as nanoparticle selection, production process, grain size, and grain boundary structure [55]. By introducing nanostructured titania fibers at 1 wt.% into the cement matrix, with the fibers acting as a reinforcing phase, an increase in resistance to brittle cracking and flexural mechanical strength was substantially improved [56]. The use of hydroxyethyl methacrylate (HEMA) for improving the affinity of TCP/PMMA blends system significantly increased the mechanical properties [57]. The mesoporous silica nanoparticles (MSNs) loaded with antibiotics showed no deterioration of mechanical properties of PMMA bone cement [58].

As regards nanometals, in [37], no negative impact of nanosilver on cement properties was observed, except for bending strength in bone cement with antibiotics. According to [34], the silver nanoparticles demonstrated no influence on the mechanical properties of the dental materials. In [39], for two cements of low and high viscosity, and a high silver content bond in bioglass, good or slightly decreased compression strength concerning the commercial cement was reported. The 0.5 wt.% of nanosilver significantly increased compressive strength [59]. The silver nanoparticles capped with tiopronin showed no effect on mechanical properties [40]. The inclusion of 0.25 wt.% of AuNPs also did not negatively alter the compressive properties of the bone cement [42]. The AgNPs at three loading ratios 0.25, 0.5, and 1.0 wt.% demonstrated mechanical properties that were not substantially different from those of the standard cement [41].

The nano-sized powders of graphene as the reinforcement of PMMA bone cements provided a significant enhancement in mechanical properties, by 12–13%, and when the graphene oxide was applied, the mechanical performance of cements was improved at low amounts of additives; over 0.25 wt.% resulted in an appearance of the agglomerates and deterioration of mechanical properties [60,61]. Improvement by 40–100% compressive and tensile strengths was obtained for PMMA with carbon nanotubes (CNTs) and reduced graphene oxide (rGO) [14]. The magnesium phosphate (MgP) nanosheets and hydroxyapatite (HA) nanofibers as novel fillers in PMMA bone cement nanocomposites also improved their compressive strength [44]. Finally, in [19] the addition of chitosan/graphene oxide nanocomposite powder to the PMMA bone cement chitosan at 25 wt.% increased the compressive strength by 16.2%, the compressive modulus by 69.1%, and the bending strength by 24.0%.

Most recently, the effects of nanoadditives have been considered with different aspects, like dispersion, degradation, corrosion, and nanomechanical properties [62]. The investigations of the effects of Cu-substituted phosphates on mechanical behavior have also not shown any detrimental effects [63].

### 1.7. Aim of the Research

The present work intends to study the behavior of bone cement with and without metallic and potentially antibacterial particles.

## 2. Materials and Methods

### 2.1. Preparation of Samples

Cemex Isoplastic cement (Tecres S.P.A., Sommacampagna, Italy) was used for the tests. Its mass fractions and chemical composition are shown in Table 1.

The tests were carried out with the addition of nanoparticles of selected metal at 1.5 wt.%. The following nanopowders were used: silver (average grain size 40 nm)—signed as Ag, copper (10–30 nm—signed as Cu 10; 70–100 nm—signed as Cu 70), silver with copper (90 nm)—signed as AgCu and nickel (10–30 nm)—signed as Ni. All powders were delivered by MKnano from Mississauga, ON, Canada.

**Figure 1:**
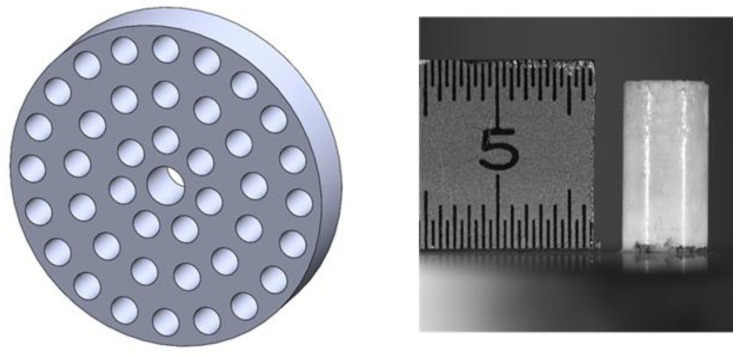
Mold to produce samples and the sample of bone cement prepared using a mold.

The cement was prepared by mixing cement powder (PMMA), liquid (hardener), and metallic powders. The mixing was performed by hand at room temperature for at least 3 min. Next, the cement was introduced in a metallic mold with 12 holes as presented in Figure 1. The mold was prepared as recommended in the ASTM F451-16 standard [64]. The whole procedure was exactly as recommended by the cement producer. The samples of 6 mm in diameter and 12 mm in length were produced with a lapse time between fabrication and testing around 24 h. After, the samples were removed from the mold. Only samples of good shape and conditions of the outside surface were selected for testing. The three samples of each type were tested and the obtained results were averaged.

### 2.2. Bacterial Testing

Bacterial tests were carried out in a broth solution (Patent No. P 409082 [65]) consisting of a nutrient medium (Table 2) and five strains of bacteria (the relative volumes shown in Table 3) mainly responsible for hospital infections. The bone cement samples were placed in the solution and examined after the 1st day and after 6-month exposure, to reflect the influence of an environment of a living organism. After this period, the samples were taken out of the broth solution to identify the bacteria with a biological microscope (Zeiss Observer D-1, Zeiss, Oberkochen, Germany), and to examine the colonization of bacteria on the surface using a scanning electron microscope (JSM-7800F, JEOL, Tokyo, Japan).

### 2.3. Cell Viability

A mammalian cell survival test was carried out to evaluate the toxicity of nanopowders added to cements. The cells of Saos-2 line (ATCC^®^ HTB-85™, LGC Standards, London, UK) were seeded in 96-well plates with McCoy’s medium supplemented with 10% of fetal bovine serum (FBS, Corning Incorporated, Corning, NY, USA) and 1% of antibiotic penicillin/streptomycin (P/S) (Corning Incorporated, Corning, NY, USA), named as full culture medium. Cells seeded at a concentration of 1 × 10^4^ cells/well were incubated for 24 h at 37 °C in 5% CO_2_ and 90% humidity. To determine the toxicity of tested nanoparticles, the protocol proposed by Satyavani et al. was applied [66], namely the prepared solutions of nanometals in full culture medium at contents of 31, 62, 125, 250, 500, and 1000 µg/mL for each type were added to wells. The tests made at increasing content reflect the anticipated situation that the nanoparticles are released from the cement in admixture into the surrounding tissue. Cells were further incubated for 24 h at 37 °C in 5% CO_2_ and 90% humidity. Cells viability was evaluated by the XTT colorimetric technique. Following a protocol, the XTT reagent (Cell Proliferation Kit XTT, AppliChem GmbH, Darmstadt, Germany) was added to each well for 4 h. Then colorimetric reading was performed using a plate microreader for 450 and 660 nm wavelengths. The results of the measured absorbance for the individually tested nanoparticles were compared to the results obtained for the control (cells only in contact with the culture medium—signed as negative control (NC)—taken as 100% viability). Each experiment was repeated three times for each type of nanometal. The relative cell viability (%) was calculated as a ratio of absorbance of a tested sample containing nanometals to the absorbance of a control sample, expressed in percentage values.

### 2.4. Contact Angle Tests

The measurements were performed with the Attension Theta Lite goniometer (Nanoscience Instruments, Phoenix, AZ, USA) with the use of a drop of distilled water at room temperature, by the falling drop technique. There were 3 samples tested of each type, the surface of each was examined in several places and the means of results and standard deviations were calculated.

### 2.5. Compressive Strength Test

The specimens for testing were prepared and the compression tests were made according to ASTM F451-16 standard with the Shimadzu AGS-X 10KN (Shimadzu Corp., Kyoto, Japan) machine at compression velocity 20 mm/min. The load and displacement were continuously recorded. The failure loading criterion was defined according to the above standard at 2% offset upper yield point or the fracture.

## 3. Results

### 3.1. Bacterial Tests

**Figure 2:**
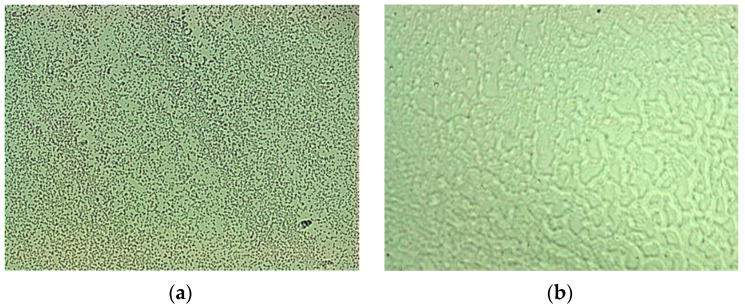
View of the solution-bacteria liquid: (**a**) after 1-day; (**b**) after 6-month. Biological microscope, 630×.

The bacteria behavior after 1 day and after 6-months exposure to bacteria liquid is shown in Figure 2a,b. After the first day, the solution contains numerous separately spread bacteria, while after 6 months the bacteria are clustered into agglomerates.

**Figure 3:**
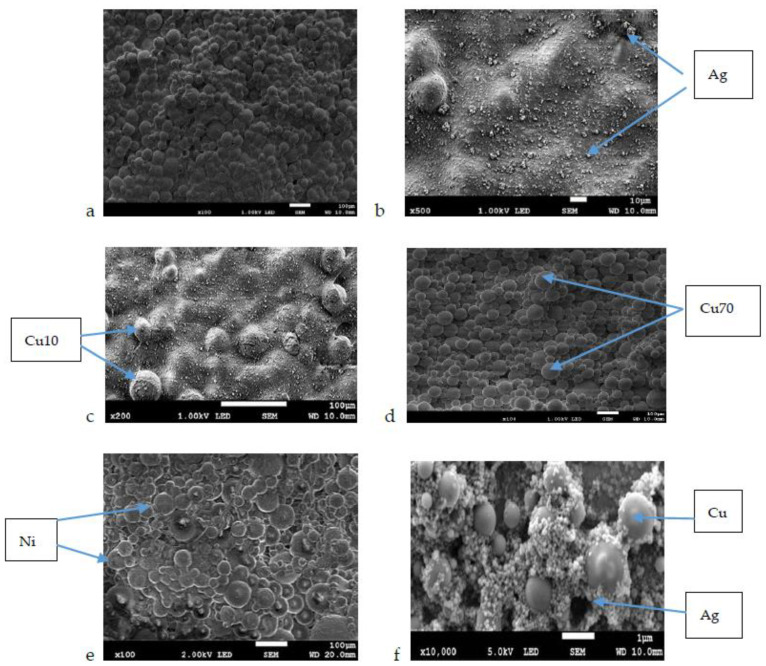
Results of the SEM evaluation of the surfaces of bone cement: (**a**) pure, and implemented with (**b**) Ag; (**c**) Cu 10; (**d**) Cu 70; (**e**) Ni; (**f**) AgCu.

Figure 3a–e illustrates the surfaces of samples of bone cement without and with added different nanometals before placing in the bacterial solution. For Cu, two different powder gradations are shown, 10 and 70 nm.

**Figure 4:**
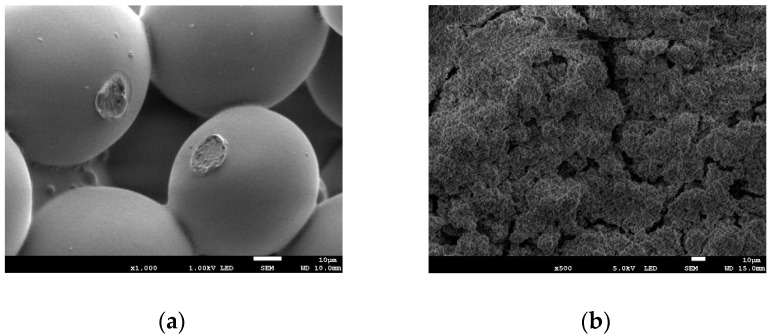

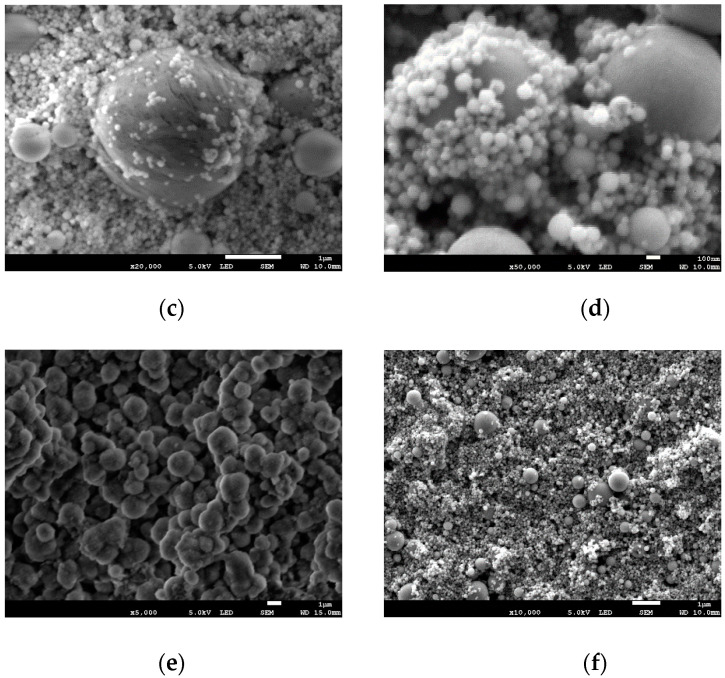
Results of the SEM evaluation of the surface of bone cement specimen after 1-day exposure in bacteria solution: (**a**) pure cement; (**b**) Ag; (**c**) Cu 10; (**d**) Cu 70; (**e**) Ni; (**f**) AgCu.

The surfaces of specimens exposed for 1 day to liquid of different bacteria are shown in Figure 4a–f. After the short-time exposure, the surfaces of all samples were free of bacteria (Figure 4a–f).

**Figure 5:**
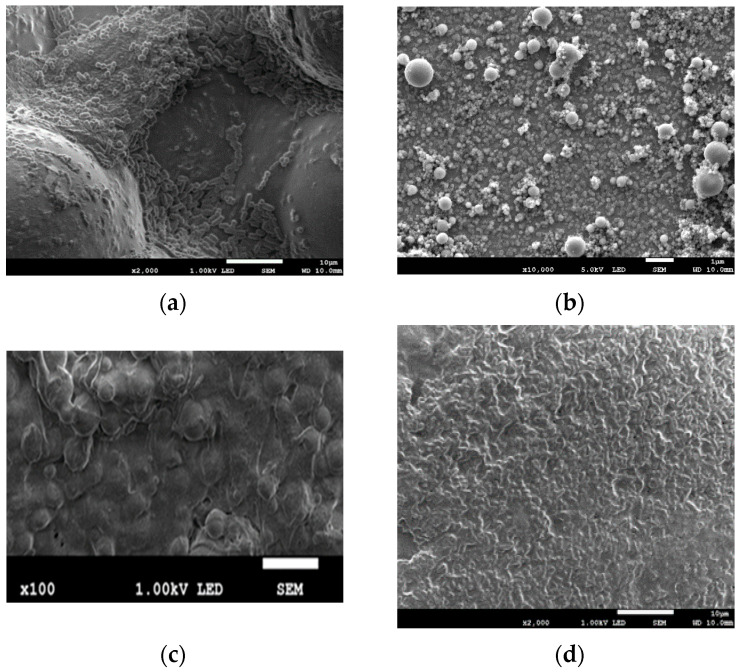

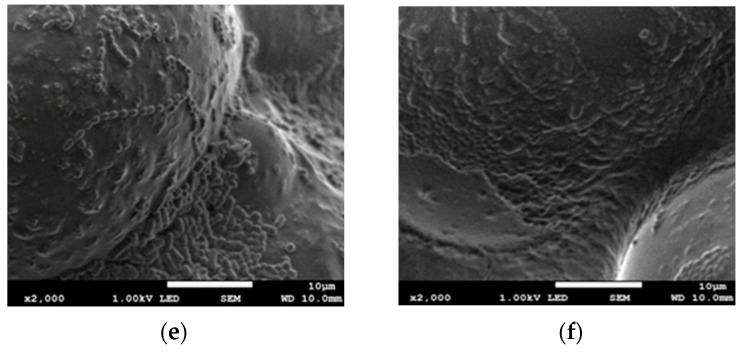
Results of the SEM evaluation of the bone cement surface after 6 months in the bacteria solution: (**a**) pure; (**b**) Ag; (**c**) Cu 10; (**d**) Cu 70; (**e**) Ni; (**f**) AgCu.

Figure 5 shows the surfaces of the samples after 6 months of their exposure. The surface of pure bone cement reveals bacteria and biofilm. Moreover, in the case of Ni and AgCu samples, bacteria adhere and biofilm is formed on the surfaces. The surface of the bone cement sample with added Ag has visible pores, without bacteria and biofilm. On both samples Cu 10 and Cu 70, there are signs of neither bacteria nor biofilm.

### 3.2. Cytotoxicity Tests

**Figure 6:**
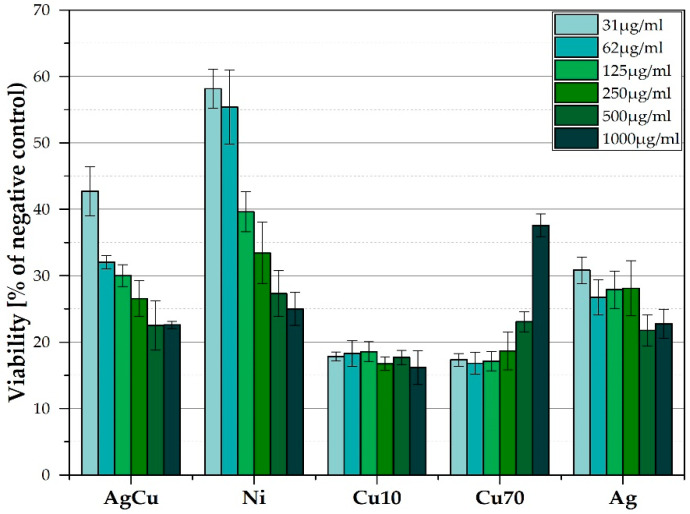
Results of the Saos-2 viability for the examined nanoparticles of silver (Ag), nickel (Ni), silver-copper (AgCu), copper with particles size in a range of 10–30 nm (Cu10), and copper with particles size in a range of 70–100 nm (Cu70).

The XTT study (Figure 6) showed that Ni nanoparticles have the most diversified impact on cell viability at 31 mg/mL content, the viability is at the level of appr. 60%, and at a maximum concentration of 1000 mg/mL, the viability is reduced by half and equals less than 30%. For silver powder, cell survival is low and remains almost at the same level regardless of the concentration—whether 31 mg/mL or 1000 mg/mL, the survival rate is between 20 and 30%. This may indicate, that more important is the mechanism of action of the silver powder on the cells rather than its quantity. For AgCu powder it can be seen that only at the lowest concentration (31 mg/mL), Cu has some effect on increasing cell survival (but only by about 10%). In the remaining concentrations, it does not neutralize the effect of Ag—the survival rate is only slightly higher than that for Ag powders—with increasing concentration of nanoparticles, the viability of cells decreases.

For Cu70 powders, the results showed the opposite relationship than for other nanoparticles—increasing powder concentration increases cells’ survival. The highest concentration of Cu70 powder (1000 mg/mL) supports cell survival—there are the most and more of them than for lower concentrations. In turn, Cu10 in the highest concentration causes the greatest reduction in cell survival of all the tested powders, and generally copper nanoparticles in sizes between 10 and 30 nm cause the lowest cell survival compared to all the tested nanoparticles. This may indicate that the grain size of the powder has an effect on the cells for this material but it is also strictly dependent on the type of material. These two factors determine the influence of nanoparticles on the biological response of the mammalian cells.

### 3.3. Wettability Tests

The contact angle measurements were carried out on samples made of pure bone cement and with nanometals. The test results are presented in Table 4.

### 3.4. Compression Tests

**Figure 7:**
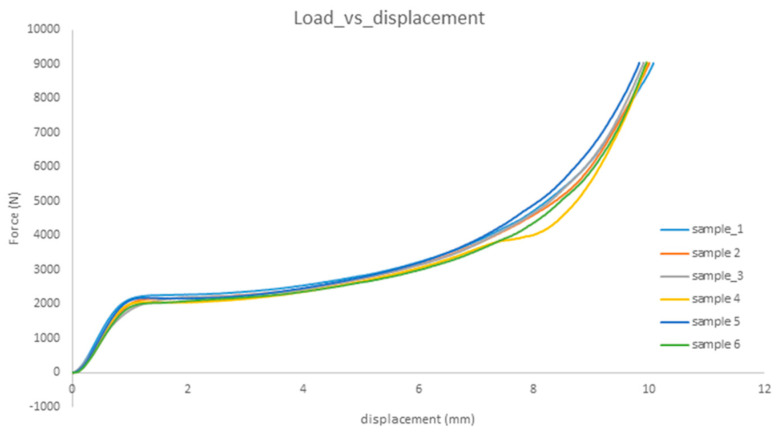
Results of force-displacement for samples of bone cement with nanoparticles; sample 1—pure bone cement; sample 2—Ag; sample 3—Cu10; sample 4—Cu70; sample 5—AgCu; sample 6—Ni.

The samples of bone cement with nanoparticles are presented in Figure 7, a single test for pure cement and each of implemented cements. The relationships are quite similar, demonstrating both linear and plastic regions.

## 4. Discussion

The present research focused on three different properties of bone cement modified by adding some nanometallic particles. These properties were chosen as essential for the application of bone cement. Antibacterial effectiveness of biomaterial is a significant property as the appearance of bacteria can be dangerous for implant fixation following the possible inflammation states. Wettability is physical property showing the potential of any material to enhance or oppose the adhesion and growth of any cells, including the growth of biofilm caused by bacteria. Finally, any addition of another component to cement may deteriorate or improve its mechanical properties, not often being a subject of testing.

### 4.1. Antibacterial Effectiveness and Cytotoxicity

As known, due to the constant increase in the resistance of bacteria to antibiotics, the nanoparticles may exceed their effectiveness [67,68], because they eliminate the problem of bacterial resistance, and also show bactericidal activity [45]. Nanoparticle carriers seem to be delivered in periods longer compared to antibiotic release [68]. For silver, its antibacterial prevention is well-known [35,36,37,38,39,40,41] and proved again by this study. The high efficiency was attained at Ag contents ranging from 0.1 to 1.0 wt.%, and here the amount of each nanometal was even higher, 1.5 wt.%. On the other side, such high content can be the reason observed here, and previously [69,70,71] slight cytotoxicity. Therefore, the necessity to maintain the nanosilver amount at a lower level, 0.25–0.5 wt.%, is confirmed. In the case of Cu nanoparticles, it is here possible to obtain a positive effect on changing the nature of this surface to a hydrophobic one, which works well in combating bacterial adhesion. However, such an effect may also result in a lack of adhesion, and thus low survival of eukaryotic cells. Since the cytotoxicity assessment tests were carried out for pure powders and 1.5% wt. pct. of each metallic additive was added to the cements, it can be assumed that, while maintaining high bactericidal activity, these materials would not show any toxic effects. It can be seen that with the decreasing concentration of the addition of Ni, Ag, and AgCu, cell survival increases. In the study of pure Cu10 and Cu70 powders, we noticed, however, that they significantly reduced the survival of Saos-2 cells. This may be related to the grain size of the nanopowder as free powder particles with a diameter of ≤32 μm, at high concentration (10^6^ particles/mL), can cause an enhanced immune response of the cells [69]. In the case of larger grain sizes, the cells surround them and try to isolate them from the environment. This neutralizes the action of the nanoparticles but does not kill the cells. For this reason, probably for the Cu70 powder (which has the largest particle size of all tested), the highest cell survival rate is observed for the highest concentration. On the other side, the possible cytotoxicity of copper nanoparticles was reported several times [68,72,73,74,75], even if it depends on the amount and size of particles. In the case of samples of cement without additives, and cement with nanoparticles of Ni and AgCu, adhering bacteria were observed on their surface after 6 months. In the case of AgCu addition, their grains are relatively large, they do not tightly fill the pores of pure cement (Figure 5f), thus creating good conditions for bacteria. So far, no studies on AgCu were revealed in the literature to compare. The Ni additive grains, although they are quite small to reduce the surface roughness, they nonetheless exhibit no antibacterial effect. In the case of this additive, studies of the powders themselves in contact with mammalian cells demonstrated that all metallic nanoparticles had a certain effect on the vitality of cells, and the addition of Ni reduced the vitality of Saos-2 cells to the smallest extent [76,77]. All these phenomena may be related to both the toxic effect of metal ions (regardless of whether they attack the cell membrane or make adhesion of bacteria easier or do not prevent it), apparently higher for copper than for silver and size of the nanoparticle, more dangerous at smaller dimensions [72,75].

### 4.2. Wettability

Research conducted by Marciano et al. [78] indicated that with the increase of the hydrophobicity of the surface, its antibacterial activity increases. This relation was also confirmed here by the tests of the contact angle and bacterial colonization. For samples Cu10 and Cu70, the contact angle was the highest, which proves their hydrophobicity. On these samples, even after 6 months of exposure to five species of bacteria, their presence was not observed. It might be then said that hydrophobic surfaces even if do not help osteoblasts to adhere, counteract the formation of biofilm. For what cells, osteoblasts or bacteria cells, this moderate hydrophobicity is more preferred, it is difficult to say without further thorough studies in the simultaneous presence of both forms of cells. The samples with Ag admixture were also free from bacteria. This is probably the effect of silver, which is well-known for its strong (the strongest among all nanomaterials) antibacterial properties. Moreover, according to some previous studies [79,80], the number of bacteria decreases when surface roughness decreases. Ag nanoparticles with a size of 40 nm fill the pores of the pure cement matrix, which reduces the surface roughness parameters and thus enhances its antibacterial properties.

### 4.3. Mechanical Behavior

Bone cement is expected to maintain its properties for a long time. An important, unfavorable feature of cement is its tendency to degrade, consisting of the loss of its original properties over time under the influence of the organism’s environment [49,81,82]. It is influenced by the aging processes of this material, additionally accelerated by the influence of the organism’s environment [83]. The phenomena typical for viscoelastic materials, including polymers, are creep and relaxation [84]. The most important factors related to the organism’s environment, that have an adverse effect on the mechanical characteristics such as static and fatigue strength fracture and creep resistance, include: cement contamination with blood and bone remains, factors related to the surgical technique, such as improper mixing and kneading techniques, delayed introduction of cement to the bone, an admixture of antibiotics and addition of a contrast agent, increased body temperature, the influence of physiological fluids (moisture and aging processes). A long time of its stay in the organism’s environment has a significant influence on the mechanical characteristics of cement [49,83,85]. During the use of artificial joints, especially hip joints, in which endoprostheses are fixed with bone cement, cement is subject to a cyclical loading process, which leads to fatigue cracking and, consequently, loosening of the endoprostheses [86,87,88,89,90]. Additionally, the greatest torsional loads affect the prosthesis [87,91,92]. The phenomenon of the destruction of the biomechanical connection of the prosthesis stem with the femur during human movement takes place under the action of cyclical load changes of high values and low frequencies, so it can be described with high probability as fatigue in the range of a small number of cycles [83].

Therefore, the chemical and phase composition of cements are important as they may affect mechanical properties. The results in these experiments of bone cement, with and without nanoparticles, are similar between samples, with a linear part and a plastic region and after with a densification region. The observed results are in accordance with a lot of earlier research for different nanomaterials. Mechanical behavior of PMMA bio-polymer was improved with some nanoadditives [90,93]. In [91], the improving various injectable materials can be obtained by using nanomaterials (Ag, Cu, Ni, AgCu) [94]. According to [16], adding fluorapatite (FA) and graphene oxide (GO) to PMMA-PCL polymer increases the mechanical properties of cement; in particular, FA increased the compressive strength and elastic modulus while reducing elongation. The modification of PMMA by carbon nanotubes increased Young’s modulus by 19% and hardness by 36% at a content of 0.15 wt.% [95]. The SiO_2_ nanoparticles improved the compression properties of PU foam, in particular at 0.5 wt% SiO_2_ elevating by 180 and 40% the compressive plateau and densification stress.

However, the nanoHA particles present in porous PU enhanced the compression resistance by 37%, but shorten the compression recovery time by 41%, and reduced the tensile resistance by 78%. In compression tests, the PMMA cement with calcium silicate retained acceptable mechanical strength and injectability [96]. On the other side, the addition of hydroxyapatite caused a decrease in the fracture toughness of nanocomposite under any stress mode [90]. The possible worsening of properties is related to element and grain size; nanocrystalline Cu had yield stresses of 450–600 MPa and strains to failure of 2–3%, but the only elastic region in nanocrystalline Ni with fractures stresses of 1200–1500 MPa, which was attributed to the grain size effect. Generally, the nanosilver caused no [34,37,40,42], or only limited [39,41] changes in mechanical properties, or even increased compression strength [59]. The present results are evidence that also other metallic nanomaterials which do not interact with cement components, have similar characteristics which scarcely depend (see results for nanocopper) on particle size in this range.

## 5. Conclusions

The antibacterial effect significantly depends on the metallic element. It is the most prominent for nanosilver and nanocopper, and negligible for AgCu and nickel nanoparticles. Most likely, the observed effects are dependent on the interaction strength and killing mechanism of metal atoms or ions with bacteria cells. The antibacterial effect is the strongest at the very beginning, after 1st day of exposure, and vanishes after 6 months of exposure for all elements except nanosilver.

The cytotoxicity appears and is similar for all investigated elements, nanosilver including. The effect may be attributed to the relatively high concentration of elements and, for nickel and copper, exceptionally strong effectiveness of killing cells, including both body cells and bacteria.

The presence of all nanometals causes the appearance of hydrophobic surfaces, more than for pure cement.

The mechanical properties are not negatively influenced by any of the elements in nanoform.

## Figures and Tables

**Table 1:** Composition of Cemex Isoplastic cement.

Liquid Components (25 wt.% of Cement)	Powder Components (75 wt.% of Cement)
Methyl methacrylate: 99.10 wt.%	Polymethyl methacrylate: 84.30 wt.%
N-N-dimetylo-p-toluidyne: 0.90 wt.%	Barium sulfate: 13.00 wt.%
Hydroquinone: 75 ppm wt.%	Benzoyl peroxide: 2.70 wt.%

**Table 2:** Composition of the bacterial medium [65].

Ingredient	Content (g/dm^3^)
Casein peptone	17
Peptone S	3
NaCl	5
Na_2_HPO_4_	2.5
Glucose	2.5

**Table 3:** Bacterial species used for the test [65].

Form	Volume Fraction (%)
*Staphylococcus aureus*	20
*Staphylococcus epidermidis*	20
*Enterococcus faecalis*	15
*Enterobacter cloacae*	10
*Pseudomonas aeruginosa*	35

**Table 4:** Water contact angle values for bone cement, pure and with nanometals.

Sample	Pure Bone Cement	Ag	Cu 10	Cu 70	Ni	AgCu
Mean value	106.29 ± 5.49	127.41 ± 5.74	139.77 ± 0.06	138.65 ± 0.13	125.71 ± 0.49	107.37 ± 5.76

## Data Availability

The data presented in this study are available on request from the corresponding author.
